# Characterization of Inner and Outer Membrane Proteins from *Francisella tularensis* Strains LVS and Schu S4 and Identification of Potential Subunit Vaccine Candidates

**DOI:** 10.1128/mBio.01592-17

**Published:** 2017-10-10

**Authors:** Deborah M. B. Post, Bram Slütter, Birgit Schilling, Aroon T. Chande, Jed A. Rasmussen, Bradley D. Jones, Alexandria K. D’Souza, Lorri M. Reinders, John T. Harty, Bradford W. Gibson, Michael A. Apicella

**Affiliations:** a Buck Institute for Research on Aging, Novato, California, USA; b Department of Microbiology, the University of Iowa, Iowa City, Iowa, USA; c Department of Pharmaceutical Chemistry, University of California, San Francisco, California, USA; University of Hawaii at Manoa

**Keywords:** *Franscisella tularensis*, immunity, inner membrane proteins, outer membrane proteins, proteomics

## Abstract

*Francisella tularensis* is the causative agent of tularemia and a potential bioterrorism agent. In the present study, we isolated, identified, and quantified the proteins present in the membranes of the virulent type A strain, Schu S4, and the attenuated type B strain, LVS (live vaccine strain). Spectral counting of mass spectrometric data showed enrichment for membrane proteins in both strains. Mice vaccinated with whole LVS membranes encapsulated in poly (lactic-co-glycolic acid) (PLGA) nanoparticles containing the adjuvant polyinosinic-polycytidylic acid [poly(I·C)] showed significant protection against a challenge with LVS compared to the results seen with naive mice or mice vaccinated with either membranes or poly(I·C) alone. The PLGA-encapsulated Schu S4 membranes with poly(I·C) alone did not significantly protect mice from a lethal intraperitoneal challenge with Schu S4; however, this vaccination strategy provided protection from LVS challenge. Mice that received the encapsulated Schu S4 membranes followed by a booster of LVS bacteria showed significant protection with respect to a lethal Schu S4 challenge compared to control mice. Western blot analyses of the sera from the Schu S4-vaccinated mice that received an LVS booster showed four immunoreactive bands. One of these bands from the corresponding one-dimensional (1D) SDS-PAGE experiment represented capsule. The remaining bands were excised, digested with trypsin, and analyzed using mass spectrometry. The most abundant proteins present in these immunoreactive samples were an outer membrane OmpA-like protein, FopA; the type IV pilus fiber building block protein; a hypothetical membrane protein; and lipoproteins LpnA and Lpp3. These proteins should serve as potential targets for future recombinant protein vaccination studies.

## IMPORTANCE

The low infectious dose, the high potential mortality/morbidity rates, and the ability to be disseminated as an aerosol make *Francisella tularensis* a potential agent for bioterrorism. These characteristics led the Centers for Disease Control (CDC) to classify *F. tularensis* as a Tier 1 pathogen. Currently, there is no vaccine approved for general use in the United States.

## INTRODUCTION

*Francisella tularensis* is a Gram-negative bacterium that is the causative agent of tularemia. Natural infection in humans can occur by contact with contaminated materials, insect bites, ingestion of contaminated water and food, or inhalation (1). The route of infection and the bacterial subspecies with which a person is infected serve as the main determining factors for the severity of disease. Some strains of *F. tularensis* are highly virulent and are able to cause disease with an infectious dose of as few as 10 organisms (2). The low infectious dose, the high potential mortality/morbidity rates, and the ability to be disseminated as an aerosol make this organism a potential agent for bioterrorism; therefore, the Centers for Disease Control and Prevention (CDC) has classified *F. tularensis* as a Tier 1 pathogen (3). However, no vaccine is currently approved for general use in the United States.

*F. tularensis* can be divided into subspecies, with two subspecies, subsp. *tularensis* (type A) and subsp. *holarctica* (type B), able to cause disease in humans. Type A strains are more virulent than type B strains. An attenuated type B strain designated the live vaccine strain (LVS) was developed in the Soviet Union and given to the United States in the 1950s (4). This vaccine has been used to immunize those at highest risk of infection, but widespread use of this vaccine was not implemented in the United States due to adverse reactions and residual virulence, and it can be presumed that the vaccine is no longer available for general use for these reasons (2, 5–7). These characteristics highlight the need for a better-defined and more effective vaccine for *F. tularensis* to be developed.

Recombinant subunit vaccines are attractive targets for the development of new *F. tularensis* vaccines because they eliminate the possibility of reversion of a live attenuated vaccine and reduce the potential toxicity associated with killed vaccines. The utilization of one or more proteins as possible subunit vaccines for *F. tularensis* has been explored by a number of groups (8). Ashtekar et al. demonstrated that the surface lipoprotein Tul4, in combination with the heat shock protein DnaK and an adjuvant, was able to provide protection against an intranasal challenge of LVS; however, the efficacy of the approach for challenge with the more virulent type A strain Schu S4 was not presented (9). FopA, an outer membrane protein (OMP), has also been shown to provide protection against a lethal challenge with LVS, but responses to this protein were not protective against a challenge with Schu S4 (10). In a different study, LVS outer membrane protein fractions emulsified in Freund’s adjuvant protected 50% of the mice that were challenged intranasally with Schu S4; however, the specific immunogenic proteins were not identified (11). More recently, Chandler et al. utilized a reverse vaccinology approach to identify a subset of 13 LVS membrane-associated proteins that were potentially immunogenic to LVS challenge (12). That study, however, did not evaluate the use of these proteins in vaccination studies, so their ability to protect against a bacterial challenge remains unknown (12). Thus, the search for a well-defined, protein-based vaccine able to protect against Schu S4 is ongoing.

In the present study, inner and outer membrane proteins were isolated from both LVS and Schu S4 strains followed by proteolytic digestion and high-resolution mass spectrometric (MS) analyses. Quantitation of the relative levels of the proteins, using weighted spectral counting, was performed to assess protein abundances in the membrane fractions (13, 14). Subsequently, mouse vaccination studies were performed using membrane fractions from LVS or Schu S4 plus polyinosinic-polycytidylic acid [poly(I·C)] that were either encapsulated in poly (lactic-co-glycolic acid) (PLGA) nanoparticles or used neat. Mice were then challenged with either LVS or Schu S4, and the antibody titers, as well as morbidity and mortality, were assessed. Immunogenic proteins were identified using a combination of Western blotting and mass spectrometric analyses. Five proteins were identified as immunogenic; four of these proteins were also highly abundant in the outer membrane of Schu S4.

## RESULTS AND DISCUSSION

### Membrane fractionation.

**FIG 1:**
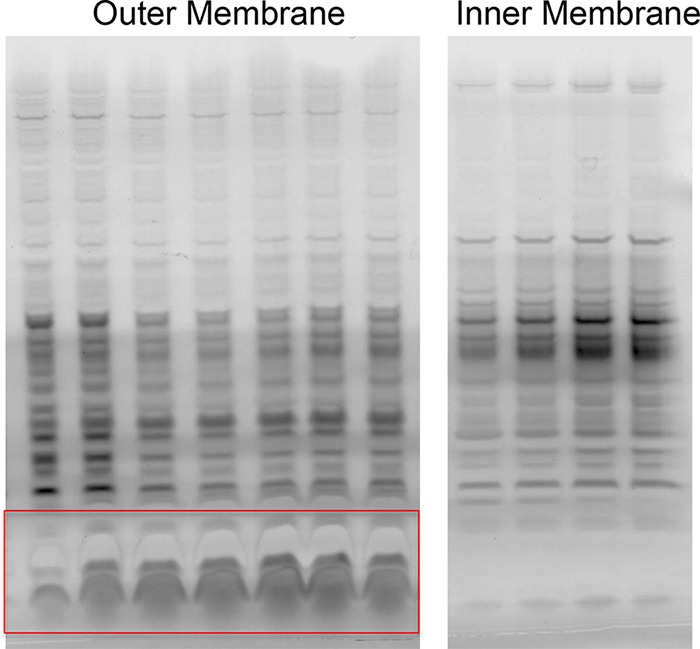
Representative results of silver-stained SDS-PAGE analysis of LVS outer and inner membrane fractions. LPS bands, shown in the red box in the outer membrane fraction panel, assisted in differentiating between the inner and outer membrane fractions.

Membranes were initially isolated from lysed Schu S4 or LVS strains of *F. tularensis* using differential centrifugation. Further separation of the membrane material was performed using a discontinuous sucrose gradient. Fractions from the sucrose gradient were collected, and absorbance readings were performed to detect which samples contained protein. The samples with protein were separated by SDS-PAGE and visualized using silver staining (Fig. 1). Fractions containing outer membrane proteins were identified by the presence of lipopolysaccharide (LPS) (Fig. 1). Inner membrane fractions were identified as the fractions that did not have lipopolysaccharide but had high protein levels.

### Analysis of inner and outer membrane profiles using liquid chromatography-tandem mass spectrometry (LC-MS/MS) and spectral counting.

In an effort to more clearly define the components of the inner and outer membranes of LVS and Schu S4, proteolytic digestion of the isolated fractions was performed. Analysis of the resulting peptides, using four injection replicates, was performed on an orthogonal time of flight mass spectrometer (TripleTOF 5600). Data from data-dependent acquisitions were searched against custom *F. tularensis* LVS or Schu S4 databases using Protein Pilot. Peptide lists from these searches were generated and filtered to include only those with at least a 99% confidence level. Weighted spectral counts were then calculated using these peptide lists (Tables S1 to S4), and proteins were ranked from highest to lowest spectral counts. These lists were then sorted from highest-ranked to lowest-ranked proteins from biological replicate 2 for all sample types.

10.1128/mBio.01592-17.5 TABLE S1  Spectral counts and rankings for three biological replicates from LVS inner membrane preparations. Download TABLE S1, XLSX file, 0.2 MB. Copyright © 2017 Post et al. 2017 Post et al. This content is distributed under the terms of the Creative Commons Attribution 4.0 International license.

The three biological replicates of the LVS inner membranes showed good reproducibility, with 16 of the top 20 proteins found in all three biological replicates and all of the top 20 proteins found in at least two of the biological replicates (Table S1). The LVS outer membrane protein samples also demonstrated good reproducibility, with 18 of the top 20 proteins found in all three biological replicates (Table S2). The top 20 proteins from the inner and outer membrane samples also showed enrichment for membrane proteins, with 16/20 proteins and 11/20 proteins, respectively, predicted to be membrane proteins, lipoproteins, or extracellular proteins. Similar spectral count rankings were seen for 12/20 proteins from the LVS inner membrane samples and 13/20 proteins from the LVS outer membrane samples. The seven proteins with the highest spectral counts, in the LVS inner membrane samples, showed consistently high spectral counts across two or more biological replicates. The proteins ranking among the top five, in the LVS outer membrane samples, had high spectral counts across all three biological replicates. The outer membrane-associated protein (Q2A2Q8; gene, FTL_1328), the 17-kDa major membrane protein (A0A0B6DRV9; gene, FTL_0421), and a hypothetical membrane protein (A0A0B3VEP0; gene, FTL_0105) were among the top-ranking proteins in all of the biological replicates of both the inner and outer membrane samples.

10.1128/mBio.01592-17.6 TABLE S2  Spectral counts and rankings for three biological replicates from LVS outer membrane preparations. Download TABLE S2, XLSX file, 0.2 MB. Copyright © 2017 Post et al. 2017 Post et al. This content is distributed under the terms of the Creative Commons Attribution 4.0 International license.

The Schu S4 inner and outer membrane protein samples showed good reproducibility, with 19 of the top 20 proteins seen in all three biological replicates (Tables S3 and S4). Inner and outer membrane samples were enriched for membrane proteins among the proteins ranking in the top 20, with 17/20 proteins and 16/20 proteins, respectively, classified as predicted membrane proteins, lipoproteins, or extracellular proteins. Similar spectral count rankings were seen for at least two biological replicates in 16/20 proteins from the Schu S4 inner membrane samples and 15/20 proteins from the Schu S4 outer membrane samples. The top five proteins for the Schu S4 inner membrane samples had high spectral counts across all three biological replicates, and the type IV pilus fiber building block protein (Q5NGF5; gene, FTT_0890c) was the top-ranked protein in two of the biological replicates and the second-highest-ranking protein in the third biological replicate. In the Schu S4 outer membrane samples, an outer membrane OmpA-like protein, FopA (Q5NH85; gene, FTT_0583), and a peptidoglycan-associated lipoprotein (Q5NGJ5; gene, FTT_0842) were among the highest-ranking proteins.

10.1128/mBio.01592-17.7 TABLE S3  Spectral counts and rankings for three biological replicates from Schu S4 inner membrane preparations. Download TABLE S3, XLSX file, 0.2 MB. Copyright © 2017 Post et al. 2017 Post et al. This content is distributed under the terms of the Creative Commons Attribution 4.0 International license.

10.1128/mBio.01592-17.8 TABLE S4  Spectral counts and rankings for three biological replicates from Schu S4 outer membrane preparations. Download TABLE S4, XLSX file, 0.2 MB. Copyright © 2017 Post et al. 2017 Post et al. This content is distributed under the terms of the Creative Commons Attribution 4.0 International license.

BLAST searches allowed us to compare the proteins identified and quantified in the LVS and Schu S4 samples (Tables S1 to S4). These alignments showed that most of the LVS and Schu S4 proteins corresponded to a homologous protein in the other strain’s database, including most of the highest-ranking membrane proteins. For example, the peptidoglycan-associated lipoprotein/outer membrane P6 domain protein (A0A0B3WIA5 and Q5NGJ5; genes, FTL_0336 and FTT_0842) was a top-20-ranked protein in the inner and outer membrane samples of both strains. Similarly, the 17-kDa membrane protein/conserved lipoprotein (A0A0B6DRV9 and Q5NGE4; genes, FTL_0421 and FTT_0901) was also a top-ranked protein in the inner and outer membrane samples of both strains. A full list of the BLAST comparisons can be found in Tables S1 to S4.

The LVS versus Schu S4 BLAST searches also showed that a small number of proteins from LVS and Schu S4 had no match in the comparison strain. For example, no match was found in the BLAST searches for one of the top-ranking proteins in the Schu S4 outer membrane, the Schu S4 type IV pilus fiber building block protein (Q5NGF5; gene, FTT_0890c). Similarly, a hypothetical protein (Q5NGE3; gene, FTT_0902) was observed in all three biological replicates of the Schu S4 inner and outer membrane samples, but the BLAST searches showed that there was no match in the LVS strain. Also, no match was found in the BLAST searches against Schu S4 with the putative outer membrane lipoprotein from LVS (Q2A4T9; gene, FTL_0491). This protein was observed in one biological replicate in both the LVS inner and outer membrane samples. The proteins that differ between the two strains may be interesting targets for future experiments.

### Formulation of membranes into nanoparticles enhances immunogenicity and protects against *F. tularensis* challenge.

**FIG 2:**
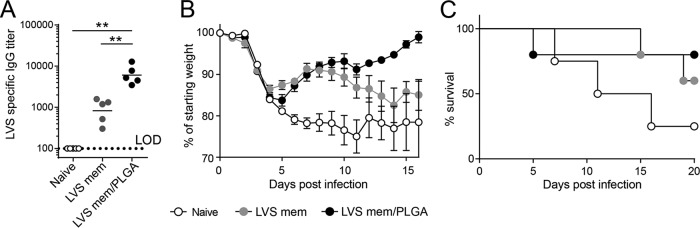
Mice were intramuscularly vaccinated with a physical mixture of 25 μg LVS membrane (LVS mem) preparations and 5 μg poly(I·C) or the same amount of LVS membranes and poly(I·C) encapsulated in PLGA nanoparticles. (A) LVS-specific IgG titers from serum 30 days postvaccination. (B and C) At 40 days postvaccination, mice were challenged with 2,000 CFU LVS intranasally and morbidity (B) and mortality (C) levels were assessed. **, *P* < 0.01 (determined by one-way ANOVA). LOD, limit of detection.

To assess vaccine potential, mice were immunized with LVS membranes in the presence of an immune potentiator [poly(I·C)]. Intramuscular immunization led to elevated membrane-specific IgG levels (Fig. 2A); however, it failed to provide significant protection against intranasal infection with LVS (Fig. 2B and C). In an effort to enhance the efficacy of the vaccine, we encapsulated the membranes into PLGA nanoparticles as encapsulation of antigens into nanoparticulate delivery systems can significantly boost the immunogenicity of subunit vaccines (15). PLGA nanoparticles are particularly well studied as vaccine delivery systems, as they have been shown to enhance B-and T-cell responses (16, 17) and have a very favorable safety profile (18). Moreover, we performed an experiment with this carrier system in which we used a Toll-like receptor (TLR) agonist adjuvant [poly(I·C)], a strategy which has been reported to enhance germinal center formation and T-cell help (19, 20). Membranes encapsulated in PLGA nanoparticles (average diameter, 286 nm) induced a significantly higher membrane-specific IgG titer than unencapsulated membranes (Fig. 2A). After intranasal challenge with a lethal dose of LVS, the majority of LVS membrane/PLGA-vaccinated mice survived the challenge (Fig. 2C) and recovered more quickly (Fig. 2B) than naive mice or mice vaccinated with membrane only. Confirming the protective role of antibodies, mice incapable of producing IgG (μS/AID doubly deficient mice [21]) were not protected from intranasal challenge with LVS after vaccination with the membrane/PLGA vaccine (see Fig. S1 in the supplemental material). Thus, LVS membranes formulated in nanoparticles protect against an otherwise lethal challenge of *F. tularensis* type B by the production of protective IgG.

10.1128/mBio.01592-17.1 FIG S1  Wild-type (C57Bl/6) and μS/AID double knockout mice were immunized with 20 μg LVS membrane and 5 μg poly(I·C) in PLGA nanoparticles. After 40 days, mice were challenged with 2,000 CFU LVS intranasally and morbidity was assessed. Download FIG S1, PDF file, 0.2 MB. Copyright © 2017 Post et al. 2017 Post et al. This content is distributed under the terms of the Creative Commons Attribution 4.0 International license.

**FIG 3:**
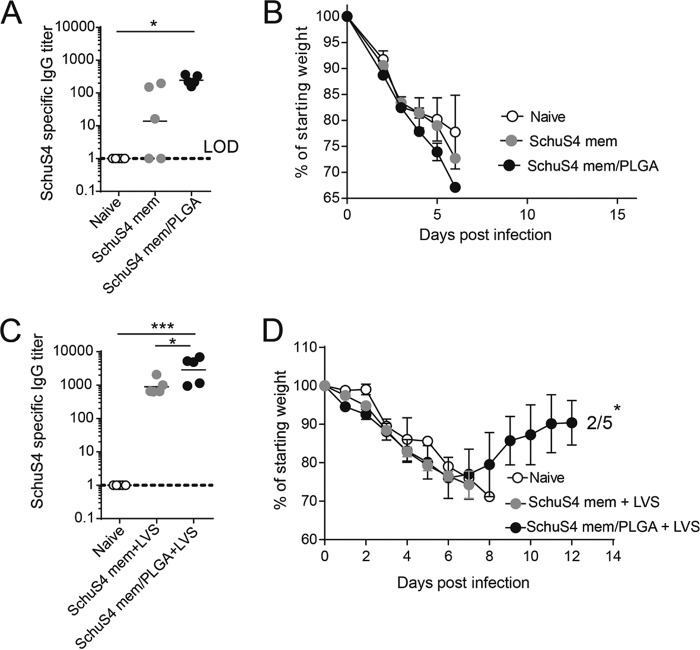
Mice were intramuscularly vaccinated with a mixture of 10 μg Schu S4 membrane preparations and 5 μg poly(I·C) or the same amount of Schu S4 membranes and poly(I·C) in PLGA nanoparticles. (A) Schu S4-specific IgG titers from serum collected 40 days postvaccination. (B) At 42 days postvaccination, mice were challenged with 25 CFU Schu S4 and morbidity and mortality levels were assessed. (C and D) Mice received a prime [mixture of 10 μg Schu S4 membranes and 5 μg poly(I·C) or the same amount of Schu S4 membrane and poly(I·C) in PLGA nanoparticles] and after 40 days received a boost (2,000 CFU LVS i.p.). (C) Schu S4-specific IgG titers from serum 37 days postboost. (D) Morbidity and mortality levels of mice were assessed. Numbers of surviving mice are indicated. *, *P* < 0.05; ***, *P* < 0.001 (determined by one-way ANOVA).

As encapsulation of membranes into PLGA nanoparticles appeared to be a successful strategy to protect against infection by a *F. tularensis* type B strain (LVS), we next sought to assess whether this immunization strategy might protect against the more virulent *F. tularensis* type A strain (Schu S4). Mice were intramuscularly immunized with Schu S4 membranes and poly(I·C) or with membranes and poly(I·C) encapsulated into PLGA nanoparticles. Again, the nanoparticulate formulation induced a higher Schu S4-specific IgG titer than the unencapsulated membranes (Fig. 3A). However, in contrast to the type B vaccine, neither of the formulations provided protection against a lethal Schu S4 challenge (Fig. 3B). Interestingly, Schu S4 membrane-vaccinated mice were very resistant to an intraperitoneal (i.p.) LVS infection (Fig. S2). In an effort to boost antibody response, we took advantage of this and boosted Schu S4 membrane-vaccinated and Schu S4 membrane/PLGA-vaccinated mice with an intraperitoneal injection of LVS. This resulted in strongly improved Schu S4-specific IgG titers (Fig. 3C). Importantly, Schu S4 membrane/PLGA-primed mice elicited significantly higher specific IgG titers than mice primed with unencapsulated Schu S4 membranes. When challenged with a lethal dose of Schu S4, a significant number of membrane/PLGA-primed, LVS-boosted mice survived the challenge (Fig. 3D), indicating that encapsulation of membranes into PLGA nanoparticles may be an interesting vaccine platform for use in combination with the existing (LVS) vaccine.

10.1128/mBio.01592-17.2 FIG S2  Mice were immunized with 10 μg Schu S4 membrane and 5 μg poly(I·C) or 10 μg Schu S4 membrane and 5 μg poly(I·C) in PLGA nanoparticles. After 40 days, mice were challenged with 2,000 CFU LVS intraperitonally and morbidity was assessed. Download FIG S2, PDF file, 0.2 MB. Copyright © 2017 Post et al. 2017 Post et al. This content is distributed under the terms of the Creative Commons Attribution 4.0 International license.

A number of different approaches have been undertaken to try and develop an effective vaccination strategy against *F. tularensis*; through these efforts, it has become clear that an effective vaccine would need to evoke a combined T-cell and B-cell protective response for efficacy (8, 22). It is likely that evoking this type of protective immune response will require the use of a live attenuated *F. tularensis* strain as part of the vaccination strategy. Our studies lend support to this conclusion, as the best survival in our Schu S4 challenge occurred when the mice were immunized with both membrane preparations and the live attenuated LVS strain. These mice were challenged with 20 times the 50% lethal dose (LD_50_) of the virulent *F. tularensis* Shu S4 strain, and 40% of the mice survived the challenge at this relatively high dose.

### Western blotting and protein identification.

**FIG 4:**
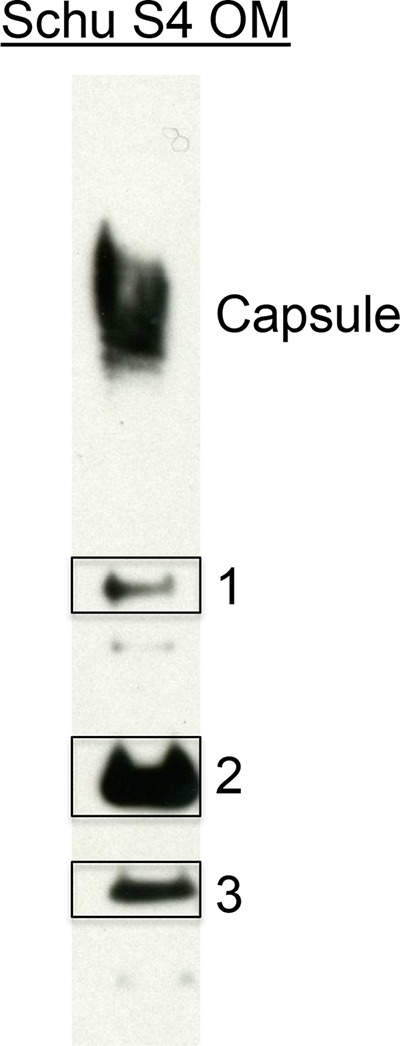
Western blot of Schu S4 outer membrane proteins probed with serum from a Schu S4 whole-membrane-PLGA-vaccinated mouse boosted with LVS obtained prior to challenge. The bands extracted for mass spectrometric analyses are indicated.

In an effort to identify the components that provided protection of LVS membrane/PLGA-vaccinated mice from an LVS challenge, Western blots of these mouse sera were probed against the OMP from LVS. These blots showed that the primary antigenic response in these mice was to capsule (Fig. S3). Western blots of Schu S4 OMP probed with sera from mice vaccinated with PLGA-encapsulated Schu S4 membranes and subsequently boosted with LVS also showed reactivity to capsule. These analyses also showed that there were three additional bands with strong reactivity to the vaccinated mouse sera (Fig. 4). These bands were extracted from a duplicate SDS-PAGE, digested with trypsin, and analyzed by high-resolution mass spectrometry. Spectral counting of the peptides, identified with at least 99% confidence, was performed (Table S5). The highest-ranked protein in gel band 1 was also the predominant protein present in the band and was identified as the outer membrane OmpA-like protein, FopA (Q5NH85; gene, FTT_0583), with a spectral count of 59.79 (Table 1). The next-highest-ranking protein in gel band 1 was the conserved hypothetical lipoprotein LpnA (Q5NGE4; gene, FTT_0901) with a spectral count of 3.21 (Table S5). The immunoreactive band 2 had two proteins with high spectral counts: a conserved lipoprotein, LpnA (Q5NGE4; gene, FTT_0901), and the type IV pilus fiber building block protein (Q5NGF5; gene, FTT_0890c), with spectral counts of 33.46 and 15.00, respectively (Table 1). The third immunoreactive band also had two proteins with high spectral counts, a hypothetical membrane protein (Q5NE75; gene, FTT_1778c) and a lipoprotein, Lpp3 (Q5NF33; gene, FTT_1416c), with spectral counts of 23.10 and 18.44, respectively (Table 1). Previous studies have suggested that FopA and the lipoproteins LpnA and Lpp3 might be possible vaccine candidates (23, 24). The genomes of approximately 50 *F. tularensis* strains have been sequenced. Using the NCBI database, BLAST analysis indicates that the five proteins described above are highly conserved among *F. tularensis* subsp. *tularensis* strains (Fig. S4). The strong immunoreactivity to and high conservation among the members of this limited set of proteins indicate that they represent attractive targets, either individually or in combination, for future vaccine development.

10.1128/mBio.01592-17.3 FIG S3  Western blot of LVS outer membrane proteins (OMP) probed with serum from LVS whole-membrane-PLGA-vaccinated mouse 6 (A) and mouse 10 (B). Lane 1, molecular mass marker (labeled in kilodaltons); lane 2, LVS OMP. Download FIG S3, PDF file, 0.02 MB. Copyright © 2017 Post et al. 2017 Post et al. This content is distributed under the terms of the Creative Commons Attribution 4.0 International license.

10.1128/mBio.01592-17.4 FIG S4  BLAST results for 10 *F. tularensis* subsp. *tularensis* strains. Download FIG S4, PDF file, 0.2 MB. Copyright © 2017 Post et al. 2017 Post et al. This content is distributed under the terms of the Creative Commons Attribution 4.0 International license.

10.1128/mBio.01592-17.9 TABLE S5  Top 10 proteins, based on spectral counts, from gel bands that reacted with sera postvaccination. Download TABLE S5, XLSX file, 0.01 MB. Copyright © 2017 Post et al. 2017 Post et al. This content is distributed under the terms of the Creative Commons Attribution 4.0 International license.

**TABLE 1:** Proteins identified from gel bands that reacted with postvaccination mouse sera

Gel band	Protein	Spectral count	Accession no.	Gene locus tag
1	Outer membrane-associated protein	59.79	Q5NH85	FTT_0583
2	Conserved hypothetical lipoprotein	33.46	Q5NGE4	FTT_0901
2	Type IV pilus fiber building block protein	15.00	Q5NGF5	FTT_0890c
3	Hypothetical membrane protein	23.10	Q5NE75	FTT_1778c
3	Hypothetical lipoprotein	18.44	Q5NF33	FTT_1416c

### Conclusions.

We have undertaken an in-depth proteomic analysis of the inner and outer membranes of *F. tularensis* LVS and *F. tularensis* Schu S4. Using sucrose density gradients, we obtained purified fractions of both membranes using the presence or absence of LPS as an indicator of the presence of the respective membrane fractions. This allowed us to obtain high-resolution proteomic analyses of both membrane fractions with little overlap. These studies indicated that the membrane proteins of the two strains were remarkably similar as our analysis identified only two proteins present in *F. tualarensis* Schu S4 which were not present in LVS, the type IV pilus fiber building block protein (Q5NGF5; gene, FTT_0890c), and a hypothetical lipoprotein (Q5NGE3; gene, FTT_0902). Our vaccination studies demonstrated that encapsulation of LVS or Schu S4 membrane particles into PLGA nanoparticles enhanced the immunogenicity of the vaccine in a *F. tularensis* LVS murine challenge model. We found that while immunization with the Schu S4 membranes in PLGA had no significant effect on survival of a Schu S4 murine intraperitoneal challenge, if we combined this with immunization with an LVS booster, a significant increase in animal survival was observed in this model. Western blots of the antigenic response to the LVS membrane/PLGA-vaccinated mice indicated that the capsule is responsible for the strongest antigenic response with this vaccination strategy. Additionally, mass spectrometric analyses of the strongest reactive bands in the Schu S4/PLGA mice that received the LVS booster identified a number of antigens previously considered possible vaccine candidates, including FopA, LpnA, and Lpp3. These data suggest that PLGA-encapsulated antigens combined with an LVS challenge may be a useful approach for protection against a virulent *F. tularensis* infection.

## MATERIALS AND METHODS

### Bacterial strains and growth conditions.

*F. tularensis* subsp. *tularensis* strain Schu S4 and *F. tularensis* subsp. *holarctica* strain LVS were grown at 37°C on chocolate agar medium supplemented with IsoVitaleX for a final cysteine concentration of 0.1%. All work with *F. tularensis* Schu S4 was performed within the Carver College of Medicine Biosafety Level 3 (BSL3) Core Facility, and experimental protocols were reviewed for safety by the BSL3 Oversight Committee of the University of Iowa Carver College of Medicine.

### Isolation of inner and outer membranes.

A complete lawn of bacteria was isolated from 20 chocolate agar plates for each *F. tularensis* strain and was suspended in a phosphate-buffered saline (PBS) solution containing cOmplete, Mini, EDTA-free protease inhibitor (Roche, Basel, Switzerland). LVS bacteria were heat killed at 65°C. Schu S4 bacteria were heat killed at 65°C, frozen at −80°C, and then heat killed at 65°C a second time to ensure that all organisms were dead prior to handling. Parallel samples were plated to ensure sterility prior to removal of specimens from the BSL3 container. Samples were individually homogenized through an Emulsiflex-C3 homogenizer three times at 15,000 lb/in^2^. Cellular debris was removed by centrifugation at 15,000 × *g* for 1 h. The supernatant was further centrifuged at 230,000 × *g* for 1.5 h. The membrane pellet from this spin was resuspended in a 50 mM HEPES solution (pH 7.2, 50 mM NaCl) and homogenized on ice in a Dounce cell grinder.

Resuspended membranes were further fractionated by diluting membrane suspensions 1:1 with a 50% sucrose solution and loading onto the top of a discontinuous sucrose gradient of 30% to 55%. Tubes were spun at ~256,000 × *g* for 20.5 h. A total of 32 fractions (approximately 0.4 ml per tube) were collected by piercing the bottom of the tubes and were dripped into sterile, capless tubes. Differentiation of the outer membrane and inner membrane fractions was performed using silver-stained SDS-PAGE. Then, the appropriate membrane fractions were pooled. Sucrose in the samples was exchanged with 100 mM Tris buffer (five times), and samples were concentrated to a final volume of ~200 μl using 3-kDa-cutoff Amicon Ultra-4 spin filters (Millipore, Billerica, MA). Three biological replicates were generated from each strain for both the inner and outer membrane samples.

### Proteolytic digestion.

The amount of protein in each pooled membrane sample was determined, and 20 μg of protein from each sample was loaded into separate lanes of a 4% to 12% SDS-PAGE gel (NuPage; Life Technologies, Inc., Carlsbad, CA). Samples were run 2 cm into the gel and visualized with Simply Blue Safe Stain (Thermo Fisher Scientific, Waltham, MA). Alternatively, samples were run on duplicate gels under normal conditions and either stained or used for Western blot analyses. Bands shown to react by Western blotting were extracted from a duplicate gel. Manual in-gel trypsin digestion was performed on all samples as previously described (25). Briefly, proteins were reduced with 10 mM dithiothreitol (DTT) and subsequently alkylated with 55 mM iodoacetamide. Proteins were digested with trypsin using a trypsin concentration of 1:20 (trypsin/protein) for 16 to 18 h at 37°C. Extracted peptides were reconstituted in a final volume of 20 μl of 1% acetonitrile–0.1% formic acid to give a concentration of 1 μg/μl. Samples were desalted with C18 Zip-tips (Millipore), according to standard procedures, prior to mass spectrometric analyses.

### Mass spectrometric analyses.

All samples were analyzed by reverse-phase high-performance liquid chromatography–electrospray ionization–tandem mass spectrometry (HPLC-ESI-MS/MS) using an Eksigent UltraPlus nano-LC two-dimensional (2D) HPLC system (Dublin, CA) connected to a quadrupole time-of-flight TripleTOF 5600 mass spectrometer (Sciex, Framingham, MA). Typically, the mass resolution for the MS1 scans and corresponding precursor ions was ~35,000 whereas the resolution for the MS2 scans and resulting fragment ions was ~15,000 (“high-sensitivity” product ion scan mode). The autosampler was operated in microliter-pickup injection mode, filling a 3-μl loop with 3 μl of analyte per injection. Briefly, after injection, peptide mixtures were transferred onto an analytical C_18_-nanocapillary HPLC column (C_18_ Acclaim PepMap100; Dionex, Sunnyvale, CA) (75-μm inner diameter [ID] by 150 mm; 3-μm particle size; 100-Å pore size) and eluted at a flow rate of 300 nl/min using the following gradient: 5% solvent B in solvent A (from 0 to 13 min), 5% to 35% solvent B in solvent A (from 13 to 58 min), 35% to 80% solvent B in solvent A (from 58 to 63 min), and 80% solvent B in solvent A (from 63 to 66 min). The elution was performed with a total run time of 90 min, including mobile-phase equilibration. Solvents were prepared as follows: for mobile phase A, 2% of acetonitrile and 98% of 0.1% (vol/vol) formic acid in water; for mobile phase B, 98% of acetonitrile and 2% of 0.1% (vol/vol) formic acid in water. Data acquisition was performed in data-dependent mode (DDA) using a TripleTOF 5600 instrument to obtain MS/MS spectra for the 30 most abundant precursor ions (approximately 50 ms per MS/MS) following each survey MS1 scan (250 ms). Each sample was analyzed in 3 biological and 4 technical injection/MS replicates. Raw data can be accessed at the MassIVE repository (https://massive.ucsd.edu; MSV000080393).

### Bioinformatic database searches.

All mass spectrometric data were searched using either ProteinPilot 4.5 (rev. 1656) running Paragon Algorithm 4.5.0.0, 1654, or ProteinPilot 5.0 (rev. 4769) running Paragon Algorithm 5.0.0.0, 4767 (SCIEX) (26), using individual custom databases for the LVS (UniProt) and Schu S4 (NCBI; AJ749949) strains. Search parameters included the following: the alkylation method used iodoacetamide; trypsin was the proteolytic enzyme; both “biological modifications” and “thorough search” were selected. The proteomics system performance evaluation pipeline (PSPEP) tool was used to generate the false-discovery-rate (FDR) analyses using a concatenated forward and reverse decoy database to search the data. The output from this analysis shows the FDR at the spectrum, peptide, and protein levels (27). In the present study, we included in our datasets only proteins with an “Unused ProtScore” value of ≥3.0, which corresponds to a protein confidence cutoff threshold of 99%. Only proteins with at least two unique peptides with ≥99% confidence were included in our final datasets. The filtered peptides lists for all biological replicates and all samples can be accessed at the MassIVE repository (https://massive.ucsd.edu; MSV000080393).

Protein localizations were predicted using the Web-based versions of SOUSI-GramN (http://harrier.nagahama-i-bio.ac.jp/sosui/sosuigramn/sosuigramn_submit.html) and PSORTb version 3.0.2 (http://www.psort.org/psortb/index.html) (28, 29) (Tables S1 to S4). BLAST searches were performed using the BLAST tool (protein BLAST) from NCBI (http://blast.ncbi.nlm.nih.gov/) and databases specified in the search summaries (Tables S1 to S4). Additional BLAST analyses were performed using Clustal W in the MegAlign suite, which is a module of Lasergene (Madison, WI).

### Spectral counting.

To estimate protein levels of the inner and outer membrane fractions, mass spectrometric acquisitions were initially subjected to two different types of spectral counting (30): (i) weighted spectral counting (13, 14), which compares the observed spectral counts for a protein to the number of theoretically possible tryptic peptides that can be generated from that protein; and (ii) normalized spectral counting, which factors in the length of the protein (31). The two methods gave similar results, and weighted spectral counting was chosen for this study (Tables S1 to S4).

### Western blot analyses.

Western blot analyses were performed as previously described by Towbin et al. (32). Samples were separated using 4% to 12% gradient gels (Invitrogen, Carlsbad, CA) and were subsequently transferred to polyvinylidene difluoride (PVDF) (Millipore). Mouse sera from challenged mice, which were either first vaccinated with PLGA encapsulated Schu S4 membranes and then with an LVS booster or were vaccinated with PLGA encapsulated LVS whole membranes, were diluted 1:5,000. Detection of the antibody was performed using a peroxidase-labeled goat anti-mouse IgM secondary antibody (Kirkegaard & Perry Laboratories, Gaithersburg, MD) and SuperSignal West Pico chemiluminescent substrate (Thermo Fisher Scientific).

### Preparation of nanoparticles.

PLGA nanoparticles were prepared using a double emulsion technique previously described (33). In short, PLGA (Sigma-Aldrich, St. Louis, MO) was dissolved in dichloromethane (2.5% [wt/vol]) and emulsified with *F. tularensis* membranes (combined inner and outer membrane preparations) and/or poly(I·C) dissolved in PBS by sonication. This mixture was emulsified with an aqueous phase containing 1% (vol/vol) Tween 20 and transferred into an excess amount of warm (>40°C) demineralized water and stirred for 1 h to harden the particles. Nanoparticles were collected by centrifugation and washed twice with PBS.

### Determining IgG titers.

High-binding microtiter plates were coated with 500 ng *F. tularensis* membranes mixed with 40 mM sodium carbonate buffer (pH 9.4) for 24 h at 4°C. Subsequently, wells were blocked with 1% (wt/vol) bovine serum albumin (BSA)–PBS for 1 h at 37°C. After extensive washing with PBS, serial dilutions of serum ranging from 20 to 2 × 10^6^ were applied and incubated for 2 h at room temperature. After extensive washing, *F. tularensis*-specific antibodies were detected using horseradish peroxidase (HRP)-conjugated goat anti-mouse IgG, IgG1, and IgG2a (Southern Biotech, Birmingham, AL) and by incubation with 0.1 mg/ml TMB (3,3',5,5'-tetramethylbenzidine)–30 μg/ml H_2_O_2_–110 mM sodium acetate buffer (pH 5.5) for 15 min at room temperature. Reactions were stopped with 2 M H_2_SO_4_, and absorbance was determined at 450 nm.

### T-cell responses.

Blood was collected by retro-orbital bleeding using heparinized capillaries, and erythrocytes were removed using ammonium chloride and washed twice. Subsequently, cells were stained with fluorescently labeled anti-CD8a, anti-CD4, anti-Thy1.2, anti-CD11a, and anti-CD49d antibodies (EBioscience, San Diego, CA) for 20 min. Flow cytometry was performed using a BD Fortezza cell analyzer.

### Mouse vaccination protocol.

For the *F. tularensis* LVS challenge experiments, groups of 5 BALB/c mice that were 6 to 8 weeks old were intramuscularly vaccinated with 25 μg of LVS membrane preparations (combined inner and outer membranes) with 5 μg poly(I·C) or with 25 μg of LVS membrane preparations with 5 μg poly(I·C) in PLGA nanoparticles. At 30 days postvaccination, blood was collected from vaccinated mice and titers of specific LVS IgG were determined. At 40 days postvaccination, mice were challenged with 2,000 CFU of the *F. tularensis* LVS strain intranasally and assessed for morbidity and mortality. To address whether protection was antibody mediated, wild-type mice (C57Bl/6) and μS/AID double knockout mice (generously provided by F. E. Lund, University of Alabama, Birmingham) were immunized with 20 μg LVS membrane and 5 μg poly(I·C) in PLGA nanoparticles. After 40 days, mice were challenged with 2,000 CFU LVS intranasally and morbidity was assessed.

For the *F. tularensis* Schu S4 challenge experiments, groups of 5 BALB/c mice that were 6 to 8 weeks old were intramuscularly vaccinated with 10 μg Schu S4 membrane preparations with 5 μg poly(I·C) or 10 μg of a Schu S4 membrane with poly(I·C) in PLGA nanoparticles. At 40 days postvaccination, blood was collected from vaccinated mice and titers of specific Schu S4 IgG were determined. At 42 days postvaccination, mice were challenged with 25 CFU of *F. tularensis* Schu S4 intraperitoneally and assessed for morbidity and mortality. As an alternative vaccination protocol, mice received intramuscularly a prime of 10 μg Schu S4 membrane preparation with 5 μg poly(I·C) or with 10 μg of a Schu S4 membrane with poly(I·C) in PLGA nanoparticles and after 40 days received a boost of 2,000 CFU of *F. tularensis* LVS delivered intraperitoneally. At 42 days postboost, mice were challenged with 25 CFU of *F. tularensis* Schu S4 intraperitoneally and assessed for morbidity and mortality. The statistical significance of the results of the antibody titer determinations and morbidity studies was determined using one-way analysis of variance (ANOVA). The statistical significance of the results of the mortality studies was determined using a Mantel-Cox (chi-square) test.

### Ethics statements.

All animals were handled in strict accordance with good animal practice as defined by the relevant national and/or local animal welfare bodies, and all animal work was approved by the University of Iowa Animal Care and Use Committee (ACURF 1305086—J. A. Rasmussen and B. D. Jones). All animal work performed with *F. tularensis* Schu S4 was performed within the Carver College of Medicine Biosafety Level 3 (BSL3) Core Facility, and all experimental protocols were reviewed for safety by the BSL3 Oversight Committee of the University of Iowa Carver College of Medicine. Guidelines provided by the NIH were followed in all experimentation. The University of Iowa is a PHS Assured institution.

## References

[1] Ellis J, Oyston PC, Green M, Titball RW 2002 Tularemia. Clin Microbiol Rev 15:631–646. doi:10.1128/CMR.15.4.631-646.2002. 12364373 PMC126859

[2] Saslaw S, Eigelsbach HT, Prior JA, Wilson HE, Carhart S 1961 Tularemia vaccine study. II. Respiratory challenge. Arch Intern Med 107:702–714. doi:10.1001/archinte.1961.03620050068007. 13746667

[3] CDC 2012 Possession, use, and transfer of select agents and toxins; biennial review. Final rule. Fed Regist 77:61083–61115. 23038847

[4] Tigertt WD 1962 Soviet viable Pasteurella tularensis vaccines. A review of selected articles. Bacteriol Rev 26:354–373. 13985026 10.1128/br.26.3.354-373.1962 PMC441156

[5] Saslaw S, Eigelsbach HT, Wilson HE, Prior JA, Carhart S 1961 Tularemia vaccine study. I. Intracutaneous challenge. Arch Intern Med 107:689–701. doi:10.1001/archinte.1961.03620050055006. 13746668

[6] Hornick RB, Eigelsbach HT 1966 Aerogenic immunization of man with live tularemia vaccine. Bacteriol Rev 30:532–538. 5917334 10.1128/br.30.3.532-538.1966 PMC378235

[7] Burke DS 1977 Immunization against tularemia: analysis of the effectiveness of live Francisella tularensis vaccine in prevention of laboratory-acquired tularemia. J Infect Dis 135:55–60. doi:10.1093/infdis/135.1.55. 833449

[8] Sunagar R, Kumar S, Franz BJ, Gosselin EJ 2016 Tularemia vaccine development: paralysis or progress? Vaccine 6:9–23. doi:10.2147/VDT.S85545. 27200274 PMC4869881

[9] Ashtekar AR, Katz J, Xu Q, Michalek SM 2012 A mucosal subunit vaccine protects against lethal respiratory infection with Francisella tularensis LVS. PLoS One 7:e50460. doi:10.1371/journal.pone.0050460. 23209745 PMC3508931

[10] Hickey AJ, Hazlett KR, Kirimanjeswara GS, Metzger DW 2011 Identification of Francisella tularensis outer membrane protein A (FopA) as a protective antigen for tularemia. Vaccine 29:6941–6947. doi:10.1016/j.vaccine.2011.07.075. 21803089 PMC3428214

[11] Huntley JF, Conley PG, Rasko DA, Hagman KE, Apicella MA, Norgard MV 2008 Native outer membrane proteins protect mice against pulmonary challenge with virulent type A Francisella tularensis. Infect Immun 76:3664–3671. doi:10.1128/IAI.00374-08. 18505805 PMC2493219

[12] Chandler JC, Sutherland MD, Harton MR, Molins CR, Anderson RV, Heaslip DG, Bosio CM, Belisle JT 2015 Francisella tularensis LVS surface and membrane proteins as targets of effective post-exposure immunization for tularemia. J Proteome Res 14:664–675. doi:10.1021/pr500628k. 25494920 PMC4324441

[13] Choi H, Fermin D, Nesvizhskii AI 2008 Significance analysis of spectral count data in label-free shotgun proteomics. Mol Cell Proteomics 7:2373–2385. doi:10.1074/mcp.M800203-MCP200. 18644780 PMC2596341

[14] Vogel C, Marcotte EM 2012 Label-free protein quantitation using weighted spectral counting. Methods Mol Biol 893:321–341. doi:10.1007/978-1-61779-885-6_20. 22665309 PMC3654649

[15] Moyer TJ, Zmolek AC, Irvine DJ 2016 Beyond antigens and adjuvants: formulating future vaccines. J Clin Invest 126:799–808. doi:10.1172/JCI81083. 26928033 PMC4767337

[16] Chong CS, Cao M, Wong WW, Fischer KP, Addison WR, Kwon GS, Tyrrell DL, Samuel J 2005 Enhancement of T helper type 1 immune responses against hepatitis B virus core antigen by PLGA nanoparticle vaccine delivery. J Control Release 102:85–99. doi:10.1016/j.jconrel.2004.09.014. 15653136

[17] Silva AL, Rosalia RA, Varypataki E, Sibuea S, Ossendorp F, Jiskoot W 2015 Poly-(lactic-co-glycolic-acid)-based particulate vaccines: particle uptake by dendritic cells is a key parameter for immune activation. Vaccine 33:847–854. doi:10.1016/j.vaccine.2014.12.059. 25576216

[18] Semete B, Booysen L, Lemmer Y, Kalombo L, Katata L, Verschoor J, Swai HS 2010 In vivo evaluation of the biodistribution and safety of PLGA nanoparticles as drug delivery systems. Nanomedicine 6:662–671. doi:10.1016/j.nano.2010.02.002. 20230912

[19] Kasturi SP, Skountzou I, Albrecht RA, Koutsonanos D, Hua T, Nakaya HI, Ravindran R, Stewart S, Alam M, Kwissa M, Villinger F, Murthy N, Steel J, Jacob J, Hogan RJ, García-Sastre A, Compans R, Pulendran B 2011 Programming the magnitude and persistence of antibody responses with innate immunity. Nature 470:543–547. doi:10.1038/nature09737. 21350488 PMC3057367

[20] Lee YR, Lee YH, Im SA, Yang IH, Ahn GW, Kim K, Lee CK 2010 Biodegradable nanoparticles containing TLR3 or TLR9 agonists together with antigen enhance MHC-restricted presentation of the antigen. Arch Pharm Res 33:1859–1866. doi:10.1007/s12272-010-1119-z. 21116790

[21] Kumazaki K, Tirosh B, Maehr R, Boes M, Honjo T, Ploegh HL 2007 AID−/−mus−/− mice are agammaglobulinemic and fail to maintain B220-CD138+ plasma cells. J Immunol 178:2192–2203. doi:10.4049/jimmunol.178.4.2192. 17277124

[22] Kirimanjeswara GS, Olmos S, Bakshi CS, Metzger DW 2008 Humoral and cell-mediated immunity to the intracellular pathogen Francisella tularensis. Immunol Rev 225:244–255. doi:10.1111/j.1600-065X.2008.00689.x. 18837786 PMC4871322

[23] Parra MC, Shaffer SA, Hajjar AM, Gallis BM, Hager A, Goodlett DR, Guina T, Miller S, Collins CM 2010 Identification, cloning, expression, and purification of Francisella lpp3: an immunogenic lipoprotein. Microbiol Res 165:531–545. doi:10.1016/j.micres.2009.11.004. 20006480

[24] Savitt AG, Mena-Taboada P, Monsalve G, Benach JL 2009 Francisella tularensis infection-derived monoclonal antibodies provide detection, protection, and therapy. Clin Vaccine Immunol 16:414–422. doi:10.1128/CVI.00362-08. 19176692 PMC2650858

[25] Post DM, Held JM, Ketterer MR, Phillips NJ, Sahu A, Apicella MA, Gibson BW 2014 Comparative analyses of proteins from Haemophilus influenzae biofilm and planktonic populations using metabolic labeling and mass spectrometry. BMC Microbiol 14:329. doi:10.1186/s12866-014-0329-9. 25551439 PMC4302520

[26] Shilov IV, Seymour SL, Patel AA, Loboda A, Tang WH, Keating SP, Hunter CL, Nuwaysir LM, Schaeffer DA 2007 The Paragon Algorithm, a next generation search engine that uses sequence temperature values and feature probabilities to identify peptides from tandem mass spectra. Mol Cell Proteomics 6:1638–1655. doi:10.1074/mcp.T600050-MCP200. 17533153

[27] Tang WH, Shilov IV, Seymour SL 2008 Nonlinear fitting method for determining local false discovery rates from decoy database searches. J Proteome Res 7:3661–3667. doi:10.1021/pr070492f. 18700793

[28] Imai K, Asakawa N, Tsuji T, Akazawa F, Ino A, Sonoyama M, Mitaku S 2008 SOSUI-GramN: high performance prediction for sub-cellular localization of proteins in gram-negative bacteria. Bioinformation 2:417–421. doi:10.6026/97320630002417. 18795116 PMC2533062

[29] Yu NY, Wagner JR, Laird MR, Melli G, Rey S, Lo R, Dao P, Sahinalp SC, Ester M, Foster LJ, Brinkman FS 2010 PSORTb 3.0: improved protein subcellular localization prediction with refined localization subcategories and predictive capabilities for all prokaryotes. Bioinformatics 26:1608–1615. doi:10.1093/bioinformatics/btq249. 20472543 PMC2887053

[30] Liu H, Sadygov RG, Yates JR III 2004 A model for random sampling and estimation of relative protein abundance in shotgun proteomics. Anal Chem 76:4193–4201. doi:10.1021/ac0498563. 15253663

[31] Zybailov B, Mosley AL, Sardiu ME, Coleman MK, Florens L, Washburn MP 2006 Statistical analysis of membrane proteome expression changes in Saccharomyces cerevisiae. J Proteome Res 5:2339–2347. doi:10.1021/pr060161n. 16944946

[32] Towbin H, Staehelin T, Gordon J 1979 Electrophoretic transfer of proteins from polyacrylamide gels to nitrocellulose sheets: procedure and some applications. Proc Natl Acad Sci U S A 76:4350–4354. doi:10.1073/pnas.76.9.4350. 388439 PMC411572

[33] Slütter B, Bal S, Keijzer C, Mallants R, Hagenaars N, Que I, Kaijzel E, van Eden W, Augustijns P, Löwik C, Bouwstra J, Broere F, Jiskoot W 2010 Nasal vaccination with N-trimethyl chitosan and PLGA based nanoparticles: nanoparticle characteristics determine quality and strength of the antibody response in mice against the encapsulated antigen. Vaccine 28:6282–6291. doi:10.1016/j.vaccine.2010.06.121. 20638455

