# Supplementary material for: Characterization of Inner and Outer Membrane Proteins from Francisella tularensis Strains LVS and Schu S4 and Identification of Potential Subunit Vaccine Candidates
Source: mBio. 2017 Oct 10;8(5):e01592-17. doi: 10.1128/mBio.01592-17 (PMC5635693; doi:10.1128/mBio.01592-17)
Supplement: FIG S1 [file mbo005173519sf1.pdf]

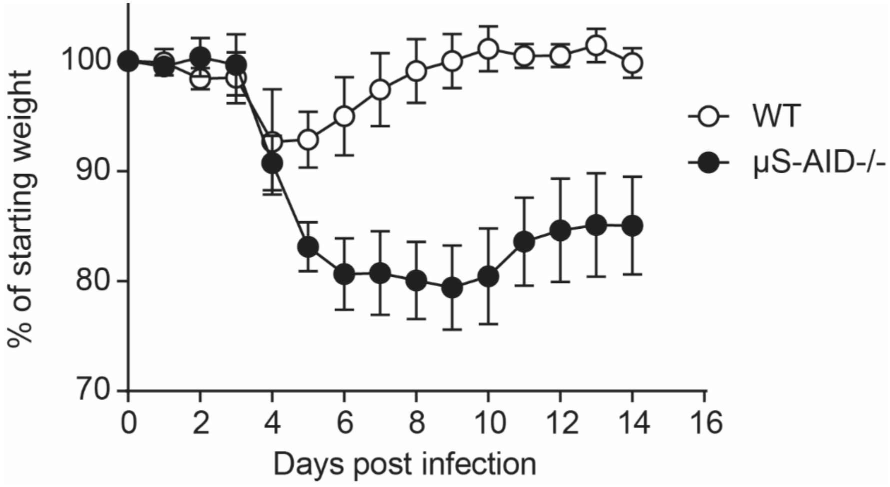

Supplemental Figure 1: Wildtype (C57Bl/6) and µS/AID double knockout mice were immunized with 20 µg LVS membrane and 5 µg PolyIC in PLGA nanoparticles. After 40 days mice were challenged with 2000 CFU LVS intranasally and morbidity was assessed.
