# Supplementary material for: Characterization of Inner and Outer Membrane Proteins from Francisella tularensis Strains LVS and Schu S4 and Identification of Potential Subunit Vaccine Candidates
Source: mBio. 2017 Oct 10;8(5):e01592-17. doi: 10.1128/mBio.01592-17 (PMC5635693; doi:10.1128/mBio.01592-17)
Supplement: FIG S2 [file mbo005173519sf2.pdf]

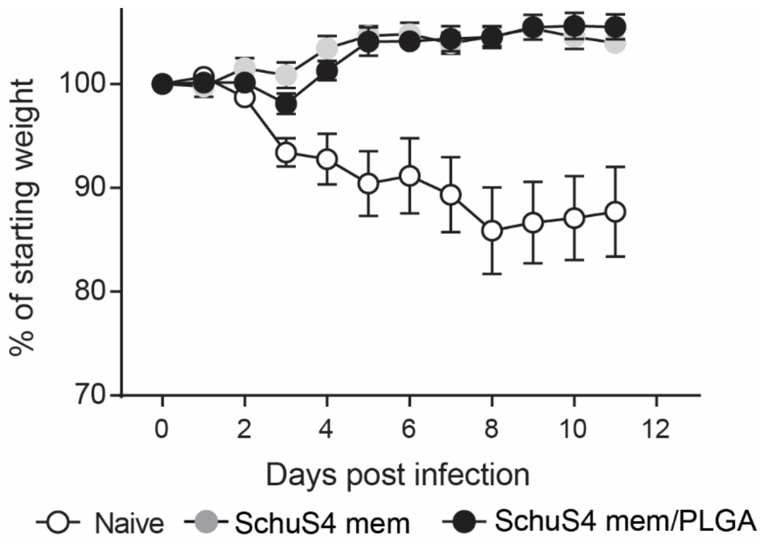

Supplemental Figure 2: Mice were immunized with 10 µg Schu S4 membrane and 5 µg PolyIC or 10 µg Schu S4 membrane and 5 µg PolyIC in PLGA nanoparticles. After 40 days mice were challenged with 2000 CFU LVS intraperitonally and morbidity was assessed.
