# Supplementary material for: Characterization of Inner and Outer Membrane Proteins from Francisella tularensis Strains LVS and Schu S4 and Identification of Potential Subunit Vaccine Candidates
Source: mBio. 2017 Oct 10;8(5):e01592-17. doi: 10.1128/mBio.01592-17 (PMC5635693; doi:10.1128/mBio.01592-17)
Supplement: FIG S3 [file mbo005173519sf3.pdf]

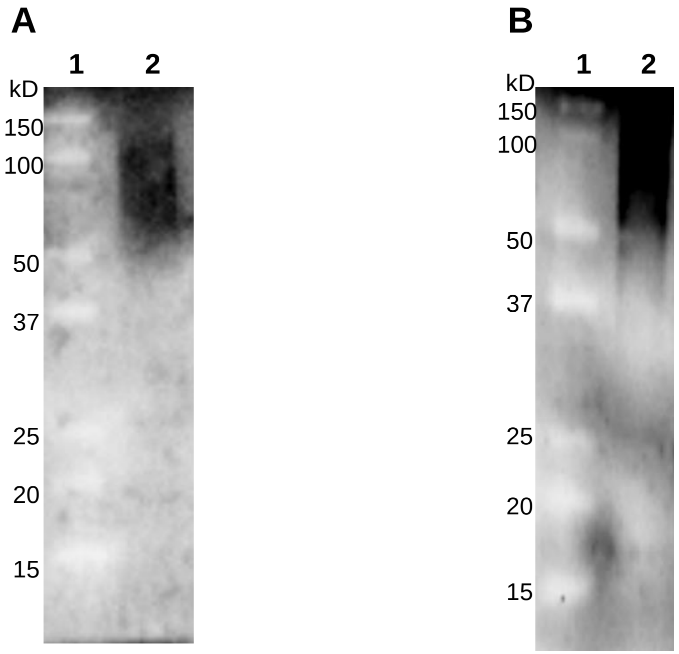

Supplemental Figure 3. Western blot of LVS outer membrane proteins (OMP) probed with serum from LVS whole membrane-PLGA vaccinated (A) mouse 6 and (B) mouse 10. Lane 1: Molecular weight marker labeled in kD and Lane 2: LVS OMP
