# Supplementary material for: Characterization of Inner and Outer Membrane Proteins from Francisella tularensis Strains LVS and Schu S4 and Identification of Potential Subunit Vaccine Candidates
Source: mBio. 2017 Oct 10;8(5):e01592-17. doi: 10.1128/mBio.01592-17 (PMC5635693; doi:10.1128/mBio.01592-17)
Supplement: FIG S4 [file mbo005173519sf4.pdf]

Supplemental Figure 4

BLAST results against 10 *F. tularensis* subsp. *tularensis* strains

FobA – Pages 1 and 2
Lpp3 – Page 3
Hypothetical Protein – Page 4
Type IV pilus Protein – Page 5
LpnA – Page 6

```
                 MMRLKSIVIATTVLLGSATASIAAGSDNIDTSANTNSATTQSSGFAANNFIAPFANTYSALTNKDNTWGP
                          10        20        30        40        50        60        70
WP_004337461. MMRLKSIVIATTVLLGSATASIAAGSDNIDTLANTNSATTQSSGFAANNFIAPFANTYSALTNKDNTWGP 70
WP_012119008. MMRLKSIVIATTVLLGSATASIAAGSDNIDTSANTNSATTQSSGFAANNFIAPFANTYSALTNKDNTWGP 70
WP_011457482. -MRLKSIVIATTVLLGSATASIAAGSDNIDTSANTNSATTQSSGFAANNFIAPFANTYSALTNKDNTWGP 69
WP_011886549. MMRLKSIVIATTVLLGSATASIAAGSDNIDTSANTNSATTQSSGFAANNFIAPFANTYSALTNKDNTWGP 70
WP_010031210. -MRLKSIVIATTVLLGSATASIAAGSDNIDTSANTNSATTQSSGFAANNFIAPFANTYSALTNKDNTWGP 69
WP_003016530. MMRLKSIVIATTVLLGSATASIAAGSDNIDTSANTNSATTQSSGFAANNFIAPFANTYSALTNKDNTWGP 70
WP_003016530. MMRLKSIVIATTVLLGSATASIAAGSDNIDTSANTNSATTQSSGFAANNFIAPFANTYSALTNKDNTWGP 70
WP_082265790. MMRLKSIVIATTVLLGSATASIAAGSDNIDTSASTNSATTQSSGFAANNFIAPFANTYSALTNKDNTWGP 70
WP_011648727. MMRLKSIVIATTVLLGSATASIAAGSDNIDTSANSNSATTQSSGFAANNFIAPFANTYSALTNKDNTWGP 70
AHH46672.1.pr -MRLKSIVIATTVLLGSATASIAAGSDNIDTSASTNSATTQSSGFAANNFIAPFANTYSALTNKDNTWGP 69

                 QDRTGQWYLGVDANGLAGTPNSPSGAGANFTIGYNINKYFAVQYNQLVGRVFAGLGEGVVNFSNNTMFTP
                          80        90       100       110       120       130       140
WP_004337461. QDRTGQWYLGVDANGLAGTPNSPSGAGANFTIGYNINKYFAVQYNQLVGRVFAGLGEGVVNFSNNTMFTP 140
WP_012119008. QDRTGQWYLGVDANGLAGTPNSPSGAGANFTIGYNINKYFAVQYNQLVGRVFAGLGEGVVNFSNNTMFTP 140
WP_011457482. QDRTGQWYLGVDANGLAGTPNSPSGAGANFTIGYNINKYFAVQYNQLVGRVFAGLGEGVVNFSNNTMFTP 139
WP_011886549. QDRTGQWYLGVDANGLARTPNSPSGAGANFTIGYNINKYFAVQYNQLVGRVFAGLGEGVVNFSNNTMFTP 140
WP_010031210. QDRTGQWYLGVDANGLAGTPNSPSGAGANFTIGYNINKYFAVQYNQLVGRVFAGLGEGVVNFSNNTMFTP 139
WP_003016530. QDRTGQWYLGVDANGLAGTPNSPSGAGANFTIGYNINKYFAVQYNQLVGRVFAGLGEGVVNFSNNTMFTP 140
WP_003016530. QDRTGQWYLGVDANGLAGTPNSPSGAGANFTIGYNINKYFAVQYNQLVGRVFAGLGEGVVNFSNNTMFTP 140
WP_082265790. QDRTGQWYLGVDANGLAGTPNSPSGAGANFTIGYNINKYFAVQYNQLVGRVFAGLGEGVVNFSNNTMFTP 140
WP_011648727. QDRTGQWYLGVDANGLAGTPNSPSGAGANFTIGYNINKYFAVQYNQLVGRVFAGLGEGVVNFSNNTMFTP 140
AHH46672.1.pr QDRTGQWYLGVDANGLAGTPNSPSGAGANFTIGYNINKYFAVQYNQLVGRVFAGLGEGVVNFSNNTMFTP 139

                 YAAGGAGWANLAGQATGAWDVGGGLKFELSRNVQASVDYRYIQTMAPSNISGANGRAGTNMIGAGLTWFF
                         150       160       170       180       190       200       210
WP_004337461. YAAGGAGWANLAGQATGAWDVGGGLKFELSRNVQASVDYRYIQTMAPSNISGANGRAGTNMIGAGLTWFF 210
WP_012119008. YAAGGAGWANLAGQATGAWDVGGGLKFELSRNVQASVDYRYIQTMAPSNISGANGRAGTNMIGAGLTWFF 210
WP_011457482. YAAGGAGWANLAGQATGAWDVGGGLKFELSRNVQASVDYRYIQTMAPSNISGANGRAGTNMIGAGLTWFF 209
WP_011886549. YAAGGAGWANLAGQATGAWDVGGGLKFELSRNVQASVDYRYIQTMAPSNISGANGRAGTNMIGAGLTWFF 210
WP_010031210. YAAGGAGWANLAGQATGAWDVGGGLKFELSRNVQASVDYRYIQTMAPSNISGANGRAGTNMIGAGLTWFF 209
WP_003016530. YAAGGAGWANLAGQATGAWDVGGGLKFELSRNVQASVDYRYIQTMAPSNISGANGRAGTNMIGAGLTWFF 210
WP_003016530. YAAGGAGWANLAGQATGAWDVGGGLKFELSRNVQASVDYRYIQTMAPSNISGANGRAGTNMIGAGLTWFF 210
WP_082265790. YAAGGAGWANLAGQATGAWDVGGGLKFELSRNVQASVDYRYIQTMAPSNISGANGRAGTNMIGAGLTWFF 210
WP_011648727. YAAGGAGWANLAGQATGAWDVGGGLKFELSRNVQASVDYRYIQTMAPSNISGANGRAGTNMIGAGLTWFF 210
AHH46672.1.pr YAAGGAGWANLAGQATGAWDVGGGLKFELSRNVQASVDYRYIQTMAPSNISGANGRAGTNMIGAGLTWFF 209

                 GGKDTTNNDTGNIQDNGATTAAQTVAMPTIDESKYVLPAGIKQCEGNFNLTEDGVACYTVNGDDVTVYLD
                         220       230       240       250       260       270       280
WP_004337461. GGKDTTNNDTGNIQDNGATTAAQTVAMPTIDESKYVLPAGIKQCEGNFNLTEDGVACYTINGDDVTVYLD 280
WP_012119008. GGKDTTNNDTGNIQDNGATTAAQTVAMPTIDESKYVLPAGIKQCEGNFNLTEDGVACYTVNGDDVTVYLD 280
WP_011457482. GGKDTTNNDTGNIQDNGATTAAQTVAMPTIDESKYVLPAGIKQCEGNFNLTEDGVACYTVNGDEVTVYLD 279
WP_011886549. GGKDTTNNDTGNIQDNGATTAAQTVAMPTIDESKYVLPAGIKQCEGNFNLTEDGVACYTVNGDDVTVYLD 280
WP_010031210. GGKDTTNNDTGNIQDNGATTAAQTVAMPTIDESKYVLPAGIKQCEGNFNLTEDGVACYTVNGDDVTVYLD 279
WP_003016530. GGKDTTNNDTGNIQDNGATTAAQTVAMPTIDESKYVLPAGIKQCEGNFNLTEDGVACYTVNGDEVTVYLD 280
WP_003016530. GGKDTTNNDTGNIQDNGATTAAQTVAMPTIDESKYVLPAGIKQCEGNFNLTEDGVACYTVNGDEVTVYLD 280
WP_082265790. GGKDTTNNDTGNIQDNGATTAAQTVAMPTIDESKYVLPAGIKQCEGNFNLTEDGVACYTVNGDDVTVYLD 280
WP_011648727. GGKDTTNNDTGNIQDNGATTAAQTVAMPTIDESKYVLPAGIKQCEGNFNLTEDGVACYTVNGDDVTVYLD 280
AHH46672.1.pr GGKDTTNNDTGNIQDNGATTAAQTVAMPTIDESKYVLPAGIKQCEGNFNLTEDGVACYTVNGDDVTVYLD 279
```

```
                TKFAYDKATLNAKGKKAIASFVNFIKDSNISSVTVKGYASQGQTGSEFDIYNQKLSEKRAQAVADYMKQL
                          290       300       310       320       330       340       350
WP_004337461.  TKFAYDKATLNAKGKKAIASFVNFIKDSNISSVTVKGYASQGQTGSEFDIYNQKLSEKRAQAVADYMKQL 350
WP_012119008.  TKFAYDKATLNAKGKKAIASFVNFIKDSNISSVTVKGYASQGQTGSEFDIYNQKLSEKRAQAVADYMKQL 350
WP_011457482.  TKFAYDKATLNAKGKKAIASFVNFIKDSNISSVTVKGYASQGQTGSEFDIYNQKLSEKRAQAVADYMKQL 349
WP_011886549.  TKFAYDKATLNAKGKKAIASFVNFIKDSNISSVTVKGYASQGQTGSEFDIYNQKLSEKRAQAVADYMKQL 350
WP_010031210.  TKFAYDKATLNAKGKKAIASFVNFIKDSNISSVTVKGYASQGQTGSEFDIYNQKLSEKRAQAVADYMKQL 349
WP_003016530.  TKFAYDKATLNAKGKKAIASFVNFIKDSNISSVTVKGYASQGQTGSEFDIYNQKLSEKRAQAVADYMKQL 350
WP_003016530.  TKFAYDKATLNAKGKKAIASFVNFIKDSNISSVTVKGYASQGQTGSEFDIYNQKLSEKRAQAVADYMKQL 350
WP_082265790.  TKFAYDKATLNAKGKKAIASFVNFIKDSNISSVTVKGYASQGQTGSEFDIYNQKLSEKRAQAVADYMKQL 350
WP_011648727.  TKFAYDKATLNAKGKKAIASFVNFIKDSNISSVTVKGYASQGQTGSEFDIYNQKLSEKRAQAVADYMKQL 350
AHH46672.1.pr  TKFAYDKATLNAKGKKAIASFVNFIKDSNISSVTVKGYASQGQTGSEFDIYNQKLSEKRAQAVADYMKQL 349

                GLDSEKIITKGFGYNDTLGGIHKSDPRNQRVEASVSAPLKEAN
                          360       370       380       390
WP_004337461.  GLDSEKIITKGFGYNDTLGGIHKSDPRNQRVEASVSAPLKEAN                            393
WP_012119008.  GLDSEKIITKGFGYNDTLGGIHKSDPRNQRVEASVSAPLKEAN                            393
WP_011457482.  GLDSEKIITKGFGYNDTLGGIHKSDPRNQRVEASVSAPLKEAN                            392
WP_011886549.  GLDSEKIITKGFGYNDTLGGIHKSDPRNQRVEASVSAPLKEAN                            393
WP_010031210.  GLDSEKIITKGFGYNDTLGGIHKSDPRNQRVEASVSAPLKEAN                            392
WP_003016530.  GLDSEKIITKGFGYNDTLGGIHKSDPRNQRVEASVSAPLKEAN                            393
WP_003016530.  GLDSEKIITKGFGYNDTLGGIHKSDPRNQRVEASVSAPLKEAN                            393
WP_082265790.  GLDSEKIITKGFGYNDTLGGIHKSDPRNQRVEASVSAPLKEAN                            393
WP_011648727.  GLDSEKIITKGFGYNDTLGGIHKSDPRNQRVEASVSAPLKEAN                            393
AHH46672.1.pr  GLDSEKIITKGFGYNDTLGGIHKSDPRNQRVEASVSAPLKEAN                            392
```

```
               MKGLKAKIYIIFLAAVLAVISGCATDKGTQYKDGYYITTLNYNFNTVYNATLQAIQNGQTFDYKSNPYDI
                        10        20        30        40        50        60        70
WP_003015055.1 MKGLKAKIYIIFLAAVLAVISGCATDKGTQYKDGYYITTLNYNFNTVYNATLQAIQNGQTFDYKSNPYDI 70
AFB79449.1.pr  MKGLKAKIYIIFLAAVLAVISGCATDKGTQYKDGYYITTLNYNFNTVYNATLQAIQNGQTFDYKSNPYDI 70
AFB80994.1.pr  MKGLKAKIYIIFLAAVLAVISGCATDKGTQYKDGYYITTLNYNFNTVYNATLQAIQNGQTFDYKSNPYDI 70
ADA79055.1.pr  MKGLKAKIYIIFLAAVLAVISGCATDKGTQYKDGYYITTLNYNFNTVYNATLQAIQNGQTFDYKSNPYDI 70
EET19742.1.pr  MKGLKAKIYIIFLAAVLAVISGCATDKGTQYKDGYYITTLNYNFNTVYNATLQAIQNGQTFDYKSNPYDI 70
EDN34981.1.pr  MKGLKAKIYIIFLAAVLAVISGCATDKGTQYKDGYYITTLNYNFNTVYNATLQAIQNGQTFDYKSNPYDI 70
ABO46387.1.pr  MKGLKAKIYIIFLAAVLAVISGCATDKGTQYKDGYYITTLNYNFNTVYNATLQAIQNGQTFDYKSNPYDI 70
CAL09432.1.pr  MKGLKAKIYIIFLAAVLAVISGCATDKGTQYKDGYYITTLNYNFNTVYNATLQAIQNGQTFDYKSNPYDI 70
CAG46049.1.pr  MKGLKAKIYIIFLAAVLAVISGCATDKGTQYKDGYYITTLNYNFNTVYNATLQAIQNGQTFDYKSNPYDI 70
YP_170359.1.p  MKGLKAKIYIIFLAAVLAVISGCATDKGTQYKDGYYITTLNYNFNTVYNATLQAIQNGQTFDYKSNPYDI 70

               SVNKNNGTDAEIVSASDSDSTDSLQVAMKKLPNNATRISIKYGSQGNSIRSSALIGIIEGNIRYANT
                        80        90       100       110       120       130
WP_003015055.1 SVNKNNGTDAEIVSASDSDSTDSLQVAMKKLPNNATRISIKYGSQGNSIRSSALIGIIEGNIRYANT      137
AFB79449.1.pr  SVNKNNGTDAEIVSASDSDSTDSLQVAMKKLPNNATRISIKYGSQGNSIRSSALIGIIEGNIRYANT      137
AFB80994.1.pr  SVNKNNGTDAEIVSASDSDSTDSLQVAMKKLPNNATRISIKYGSQGNSIRSSALIGIIEGNIRYANT      137
ADA79055.1.pr  SVNKNNGTDAEIVSASDSDSTDSLQVAMKKLPNNATRISIKYGSQGNSIRSSALIGIIEGNIRYANT      137
EET19742.1.pr  SVNKNNGTDAEIVSASDSDSTDSLQVAMKKLPNNATRISIKYGSQGNSIRSSALIGIIEGNIRYANT      137
EDN34981.1.pr  SVNKNNGTDAEIVSASDSDSTDSLQVAMKKLPNNATRISIKYGSQGNSIRSSALIGIIEGNIRYANT      137
ABO46387.1.pr  SVNKNNGTDAEIVSASDSDSTDSLQVAMKKLPNNATRISIKYGSQGNSIRSSALIGIIEGNIRYANT      137
CAL09432.1.pr  SVNKNNGTDAEIVSASDSDSTDSLQVAMKKLPNNATRISIKYGSQGNSIRSSALIGIIEGNIRYANT      137
CAG46049.1.pr  SVNKNNGTDAEIVSASDSDSTDSLQVAMKKLPNNATRISIKYGSQGNSIRSSALIGIIEGNIRYANT      137
YP_170359.1.p  SVNKNNGTDAEIVSASDSDSTDSLQVAMKKLPNNATRISIKYGSQGNSIRSSALIGIIEGNIRYANT      137
```

```
              MKLRKVLIATLLGASALSLSSCWLLVGAAVGGGTAAYISGEYSMNMSGSVKDIYNATLKAVQSNDDFVIT
                       10        20        30        40        50        60        70
WP_003017667. MKLRKVLIATLLGASALSLSSCWLLVGAAVGGGTAAYISGEYSMNMSGSVKDIYNATLKAVQSNDDFVIT 70
ABO46021.1.pr MKLRKVLIATLLGASALSLSSCWLLVGAAVGGGTAAYISGEYSMNMSGSVKDIYNATLKAVQSNDDFVIT 70
EKM89467.1.pr MKLRKVLIATLLGASALSLSSCWLLVGAAVGGGTAAYISGEYSMNMSGSVKDIYNATLKAVQSNDDFVIT 70
EKM89495.1.pr MKLRKVLIATLLGASALSLSSCWLLVGAAVGGGTAAYISGEYSMNMSGSVKDIYNATLKAVQSNDDFVIT 70
EKM92566.1.pr MKLRKVLIATLLGASALSLSSCWLLVGAAVGGGTAAYISGEYSMNMSGSVKDIYNATLKAVQSNDDFVIT 70
EKM93460.1.pr MKLRKVLIATLLGASALSLSSCWLLVGAAVGGGTAAYISGEYSMNMSGSVKDIYNATLKAVQSNDDFVIT 70
EKT90622.1.pr MKLRKVLIATLLGASALSLSSCWLLVGAAVGGGTAAYISGEYSMNMSGSVKDIYNATLKAVQSNDDFVIT 70
EMI60337.1.pr MKLRKVLIATLLGASALSLSSCWLLVGAAVGGGTAAYISGEYSMNMSGSVKDIYNATLKAVQSNDDFVIT 70
AJI63838.1.pr MKLRKVLIATLLGASALSLSSCWLLVGAAVGGGTAAYISGEYSMNMSGSVKDIYNATLKAVQSNDDFVIT 70
AKH91139.1.pr MKLRKVLIATLLGASALSLSSCWLLVGAAVGGGTAAYISGEYSMNMSGSVKDIYNATLKAVQSNDDFVIT 70

              KKSITSVDAVVDGSTKVDSTSFYVKIEKLTDNASKVTIKFGTFGDQAMSATLMDQIQKNL
                       80        90       100       110       120       130
WP_003017667. KKSITSVDAVVDGSTKVDSTSFYVKIEKLTDNASKVTIKFGTFGDQAMSATLMDQIQKNL           130
ABO46021.1.pr KKSITSVDAVVDGSTKVDSTSFYVKIEKLTDNASKVTIKFGTFGDQAMSATLMDQIQKNL           130
EKM89467.1.pr KKSITSVDAVVDGSTKVDSTSFYVKIEKLTDNASKVTIKFGTFGDQAMSATLMDQIQKNL           130
EKM89495.1.pr KKSITSVDAVVDGSTKVDSTSFYVKIEKLTDNASKVTIKFGTFGDQAMSATLMDQIQKNL           130
EKM92566.1.pr KKSITSVDAVVDGSTKVDSTSFYVKIEKLTDNASKVTIKFGTFGDQAMSATLMDQIQKNL           130
EKM93460.1.pr KKSITSVDAVVDGSTKVDSTSFYVKIEKLTDNASKVTIKFGTFGDQAMSATLMDQIQKNL           130
EKT90622.1.pr KKSITSVDAVVDGSTKVDSTSFYVKIEKLTDNASKVTIKFGTFGDQAMSATLMDQIQKNL           130
EMI60337.1.pr KKSITSVDAVVDGSTKVDSTSFYVKIEKLTDNASKVTIKFGTFGDQAMSATLMDQIQKNL           130
AJI63838.1.pr KKSITSVDAVVDGSTKVDSTSFYVKIEKLTDNASKVTIKFGTFGDQAMSATLMDQIQKNL           130
AKH91139.1.pr KKSITSVDAVVDGSTKVDSTSFYVKIEKLTDNASKVTIKFGTFGDQAMSATLMDQIQKNL           130
```

```
                 MKKKMQKGFSLVELMVVIAIIAILAAVAIPMYSNYTTRAQLGSDLSALGGAKATVAERIANNNGDASQVT
                          10        20        30        40        50        60        70
WP_003020882.    MKKKMQKGFSLVELMVVIAIIAILAAVAIPMYSNYTTRAQLGSDLSALGGAKATVAERIANNNGDASQVT 70
ABK78954.1.pr    MKKKMQKGFSLVELMVVIAIIAILAAVAIPMYSNYTTRAQLGSDLSALGGAKATVAERIANNNGDASQVT 70
ABK78953.1.pr    MKKKMQKGFSLVELMVVIAIIAILAAVAIPMYSNYTTRAQLGSDLSALGGAKATVAERIANNNGDASQVT 70
ABK78952.1.pr    MKKKMQKGFSLVELMVVIAIIAILAAVAIPMYSNYTTRAQLGSDLSALGGAKATVAERIANNNGDASQVT 70
ABK78951.1.pr    MKKKMQKGFSLVELMVVIAIIAILAAVAIPMYSNYTTRAQLGSDLSALGGAKATVAERIANNNGDASQVT 70
ABK78950.1.pr    MKKKMQKGFSLVELMVVIAIIAILAAVAIPMYSNYTTRAQLGSDLSALGGAKATVAERIANNNGDASQVT 70
ABK78949.1.pr    MKKKMQKGFSLVELMVVIAIIAILAAVAIPMYSNYTTRAQLGSDLSALGGAKATVAERIANNNGDASQVT 70
CAL08906.1.pr    MKKKMQKGFSLVELMVVIAIIAILAAVAIPMYSNYTTRAQLGSDLSALGGAKATVAERIANNNGDASQVT 70
CAG45523.1.pr    MKKKMQKGFSLVELMVVIAIIAILAAVAIPMYSNYTTRAQLGSDLSALGGAKATVAERIANNNGDASQVT 70
YP_169887.1.p    MKKKMQKGFSLVELMVVIAIIAILAAVAIPMYSNYTTRAQLGSDLSALGGAKATVAERIANNNGDASQVT 70

                 ILQANAAANGLPSGASVAAGTISYPSTVSGATIQLAPTVSSGAITWTCNISGVSASQVPSNCNAI
                          80        90       100       110       120       130
WP_003020882.    ILQANAAANGLPSGASVAAGTISYPSTVSGATIQLAPTVSSGAITWTCNISGVSASQVPSNCNAI      135
ABK78954.1.pr    ILQANAAANGLPSGASVAAGTISYPSTVSGATIQLAPTVSSGAITWTCNISGVSASQVPSNCNAI      135
ABK78953.1.pr    ILQANAAANGLPSGASVAAGTISYPSTVSGATIQLAPTVSSGAITWTCNISGVSASQVPSNCNAI      135
ABK78952.1.pr    ILQANAAANGLPSGASVAAGTISYPSTVSGATIQLAPTVSSGAITWTCNISGVSASQVPSNCNAI      135
ABK78951.1.pr    ILQANAAANGLPSGASVAAGTISYPSTVSGATIQLAPTVSSGAITWTCNISGVSASQVPSNCNAI      135
ABK78950.1.pr    ILQANAAANGLPSGASVAAGTISYPSTVSGATIQLAPTVSSGAITWTCNISGVSASQVPSNCNAI      135
ABK78949.1.pr    ILQANAAANGLPSGASVAAGTISYPSTVSGATIQLAPTVSSGAITWTCNISGVSASQVPSNCNAI      135
CAL08906.1.pr    ILQANAAANGLPSGASVAAGTISYPSTVSGATIQLAPTVSSGAITWTCNISGVSASQVPSNCNAI      135
CAG45523.1.pr    ILQANAAANGLPSGASVAAGTISYPSTVSGATIQLAPTVSSGAITWTCNISGVSASQVPSNCNAI      135
YP_169887.1.p    ILQANAAANGLPSGASVAAGTISYPSTVSGATIQLAPTVSSGAITWTCNISGVSASQVPSNCNAI      135
```

```
                MKKIIELSLLSLSIAGLASCSTLGLGGSDDAKASAKDTAAAQTATTEQAAAVSKPTAKVSLNKLGQDKIK
                         10        20        30        40        50        60        70
WP_003020907.   MKKIIELSLLSLSIAGLASCSTLGLGGSDDAKASAKDTAAAQTATTEQAAAVSKPTAKVSLNKLGQDKIK 70
AFB78998.1.pr   MKKIIELSLLSLSIAGLASCSTLGLGGSDDAKASAKDTAAAQTATTEQAAAVSKPTAKVSLNKLGQDKIK 70
AFB80543.1.pr   MKKIIELSLLSLSIAGLASCSTLGLGGSDDAKASAKDTAAAQTATTEQAAAVSKPTAKVSLNKLGQDKIK 70
ADA78581.1.pr   MKKIIELSLLSLSIAGLASCSTLGLGGSDDAKASAKDTAAAQTATTEQAAAVSKPTAKVSLNKLGQDKIK 70
EET19237.1.pr   MKKIIELSLLSLSIAGLASCSTLGLGGSDDAKASAKDTAAAQTATTEQAAAVSKPTAKVSLNKLGQDKIK 70
EDN34382.1.pr   MKKIIELSLLSLSIAGLASCSTLGLGGSDDAKASAKDTAAAQTATTEQAAAVSKPTAKVSLNKLGQDKIK 70
ABO47056.1.pr   MKKIIELSLLSLSIAGLASCSTLGLGGSDDAKASAKDTAAAQTATTEQAAAVSKPTAKVSLNKLGQDKIK 70
CAL08917.1.pr   MKKIIELSLLSLSIAGLASCSTLGLGGSDDAKASAKDTAAAQTATTEQAAAVSKPTAKVSLNKLGQDKIK 70
CAG45534.1.pr   MKKIIELSLLSLSIAGLASCSTLGLGGSDDAKASAKDTAAAQTATTEQAAAVSKPTAKVSLNKLGQDKIK 70
YP_169898.1.p   MKKIIELSLLSLSIAGLASCSTLGLGGSDDAKASAKDTAAAQTATTEQAAAVSKPTAKVSLNKLGQDKIK 70

                ATVYTTYNNNPQGSVRLQWQAPEGSKCHDTSFPITKYAEKNDKTWATVTVKQGNNFCSGKWTANVVYDKE
                         80        90        100       110       120       130       140
WP_003020907.   ATVYTTYNNNPQGSVRLQWQAPEGSKCHDTSFPITKYAEKNDKTWATVTVKQGNNFCSGKWTANVVYDKE 140
AFB78998.1.pr   ATVYTTYNNNPQGSVRLQWQAPEGSKCHDTSFPITKYAEKNDKTWATVTVKQGNNFCSGKWTANVVYDKE 140
AFB80543.1.pr   ATVYTTYNNNPQGSVRLQWQAPEGSKCHDTSFPITKYAEKNDKTWATVTVKQGNNFCSGKWTANVVYDKE 140
ADA78581.1.pr   ATVYTTYNNNPQGSVRLQWQAPEGSKCHDTSFPITKYAEKNDKTWATVTVKQGNNFCSGKWTANVVYDKE 140
EET19237.1.pr   ATVYTTYNNNPQGSVRLQWQAPEGSKCHDTSFPITKYAEKNDKTWATVTVKQGNNFCSGKWTANVVYDKE 140
EDN34382.1.pr   ATVYTTYNNNPQGSVRLQWQAPEGSKCHDTSFPITKYAEKNDKTWATVTVKQGNNFCSGKWTANVVYDKE 140
ABO47056.1.pr   ATVYTTYNNNPQGSVRLQWQAPEGSKCHDTSFPITKYAEKNDKTWATVTVKQGNNFCSGKWTANVVYDKE 140
CAL08917.1.pr   ATVYTTYNNNPQGSVRLQWQAPEGSKCHDTSFPITKYAEKNDKTWATVTVKQGNNFCSGKWTANVVYDKE 140
CAG45534.1.pr   ATVYTTYNNNPQGSVRLQWQAPEGSKCHDTSFPITKYAEKNDKTWATVTVKQGNNFCSGKWTANVVYDKE 140
YP_169898.1.p   ATVYTTYNNNPQGSVRLQWQAPEGSKCHDTSFPITKYAEKNDKTWATVTVKQGNNFCSGKWTANVVYDKE 140

                VIASDSINI

WP_003020907.   VIASDSINI                                                              149
AFB78998.1.pr   VIASDSINI                                                              149
AFB80543.1.pr   VIASDSINI                                                              149
ADA78581.1.pr   VIASDSINI                                                              149
EET19237.1.pr   VIASDSINI                                                              149
EDN34382.1.pr   VIASDSINI                                                              149
ABO47056.1.pr   VIASDSINI                                                              149
CAL08917.1.pr   VIASDSINI                                                              149
CAG45534.1.pr   VIASDSINI                                                              149
YP_169898.1.p   VIASDSINI                                                              149
```
